# Supplementary figures and images for: Harnessing fluorescent carbon quantum dots from natural resource for advancing sweat latent fingerprint recognition with machine learning algorithms for enhanced human identification
Source: PLoS One. 2024 Jan 4;19(1):e0296270. doi: 10.1371/journal.pone.0296270 (PMC10766178; doi:10.1371/journal.pone.0296270)

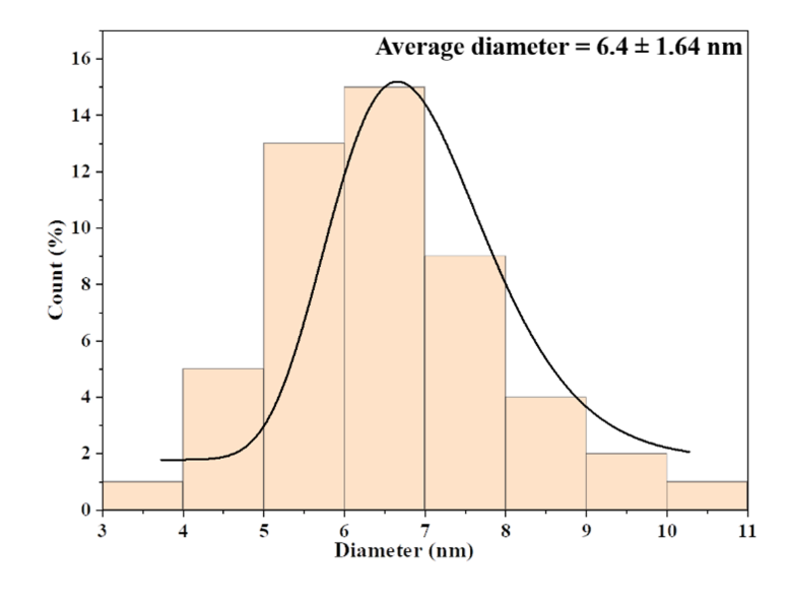

Supplement: S1 Fig — (TIF) [file pone.0296270.s001.tif]

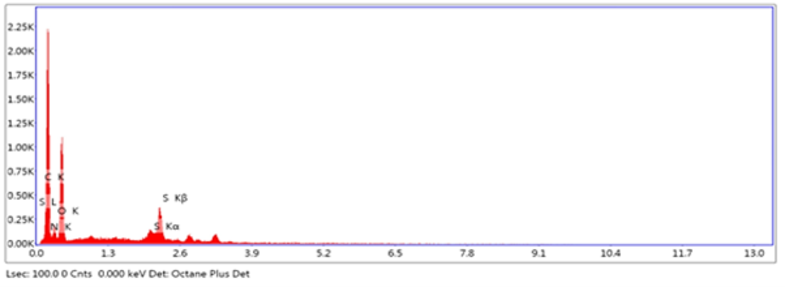

Supplement: S2 Fig — (TIF) [file pone.0296270.s002.tif]

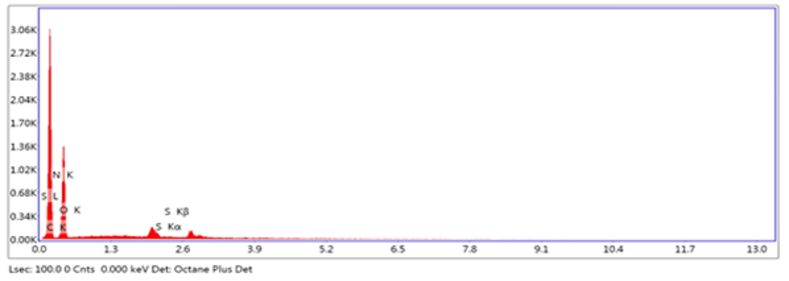

Supplement: S3 Fig — (TIF) [file pone.0296270.s003.tif]

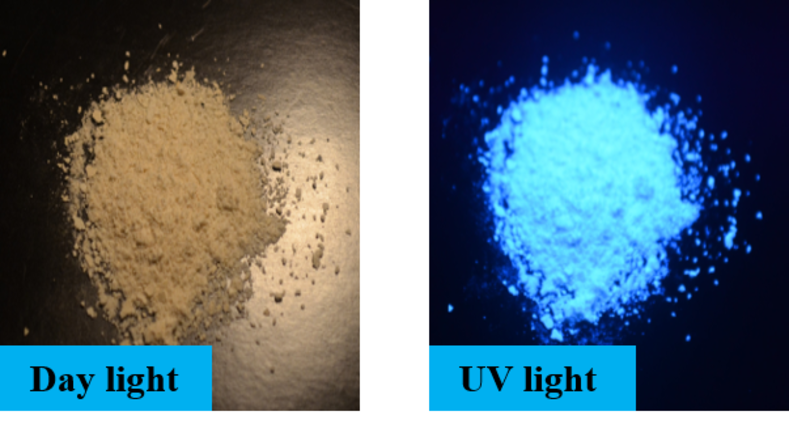

Supplement: S4 Fig — (TIF) [file pone.0296270.s004.tif]

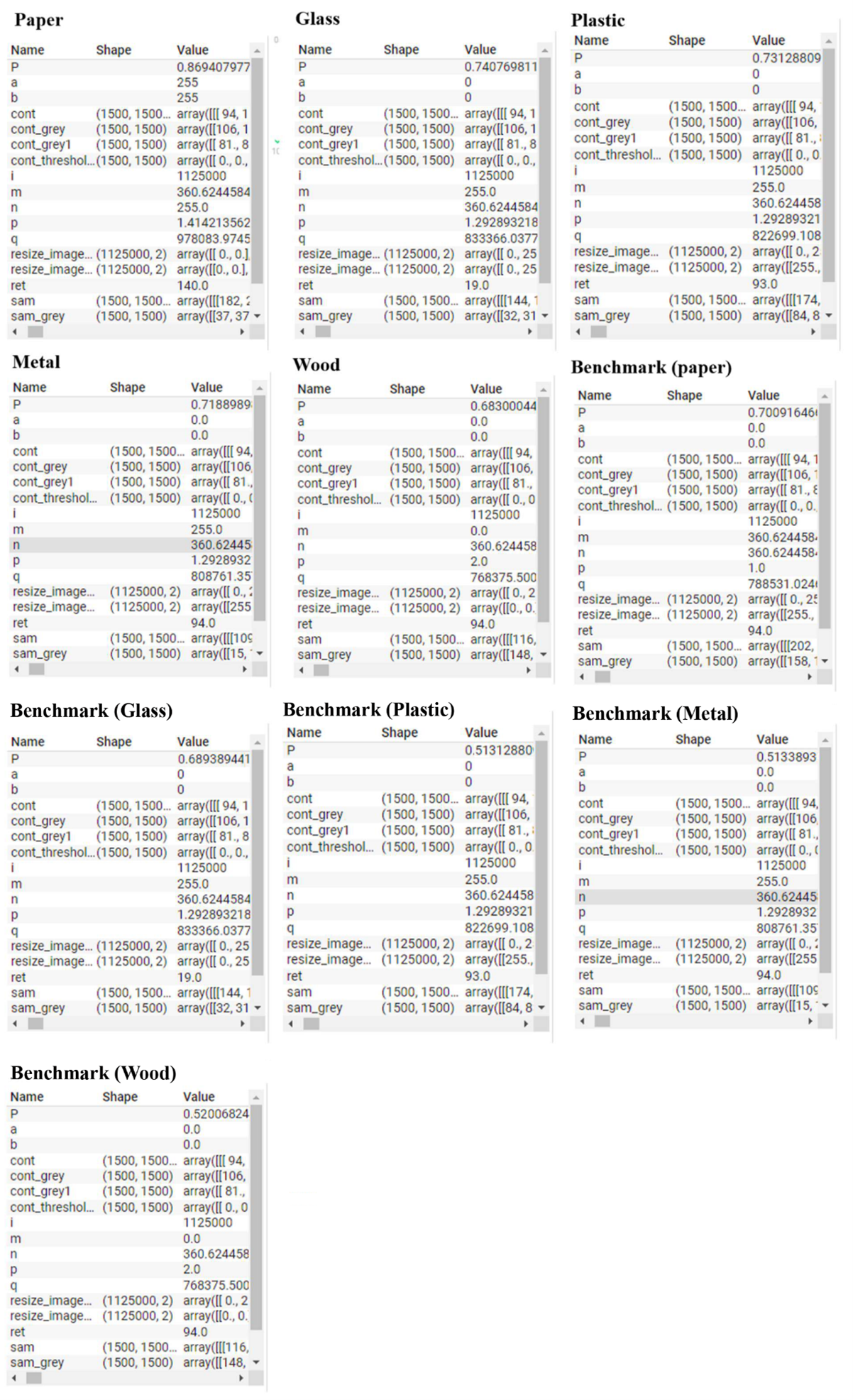

Supplement: S5 Fig — (TIF) [file pone.0296270.s005.tif]

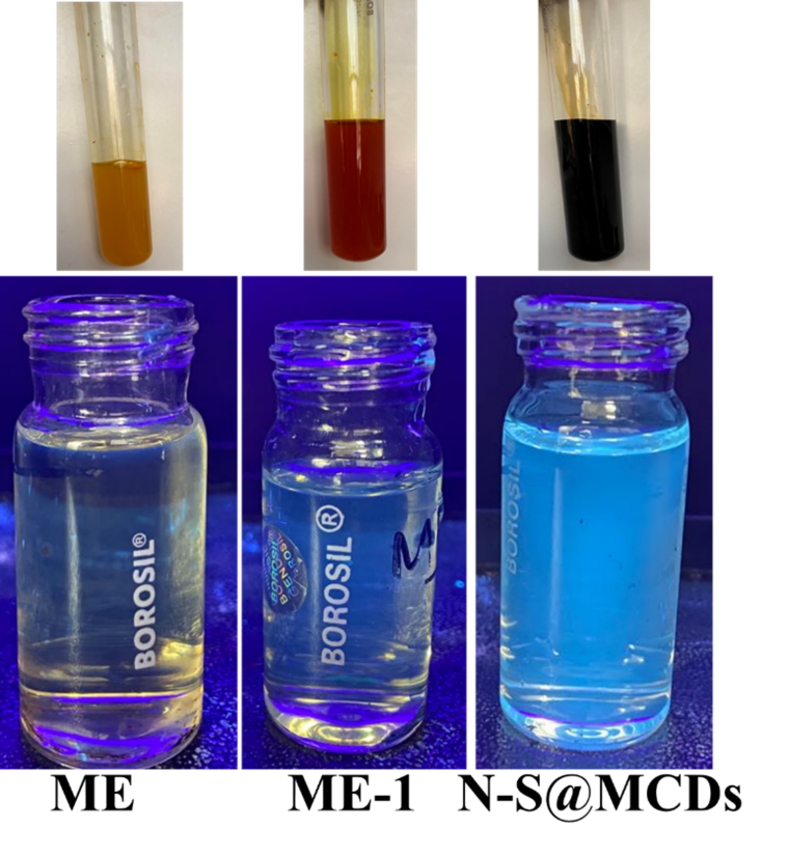

Supplement: S6 Fig — (TIF) [file pone.0296270.s006.tif]

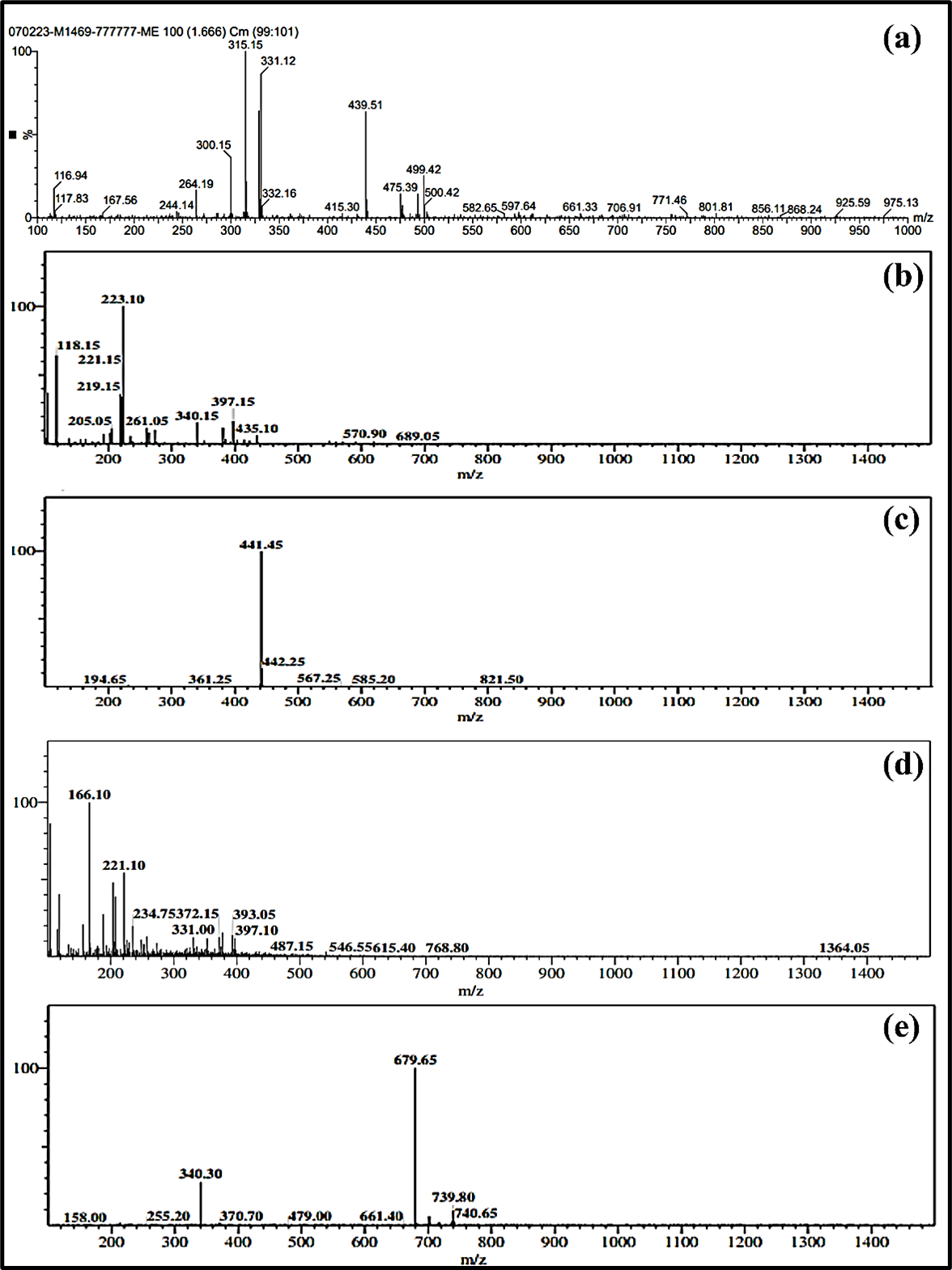

Supplement: S7 Fig — LC-MS chromatogram of ME (a), ME-1 (b & c) and N-S@MCDs (d &e). (TIF) [file pone.0296270.s007.tif]

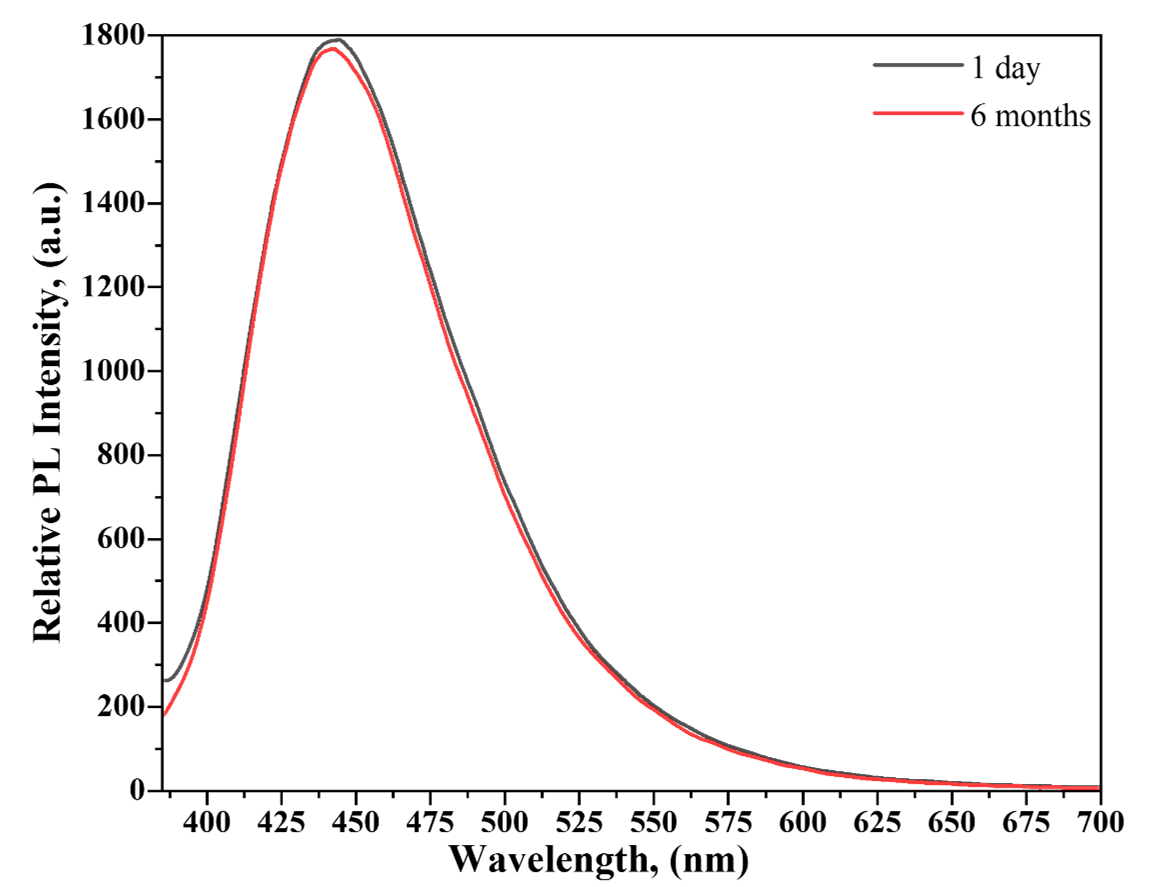

Supplement: S8 Fig — (TIF) [file pone.0296270.s008.tif]

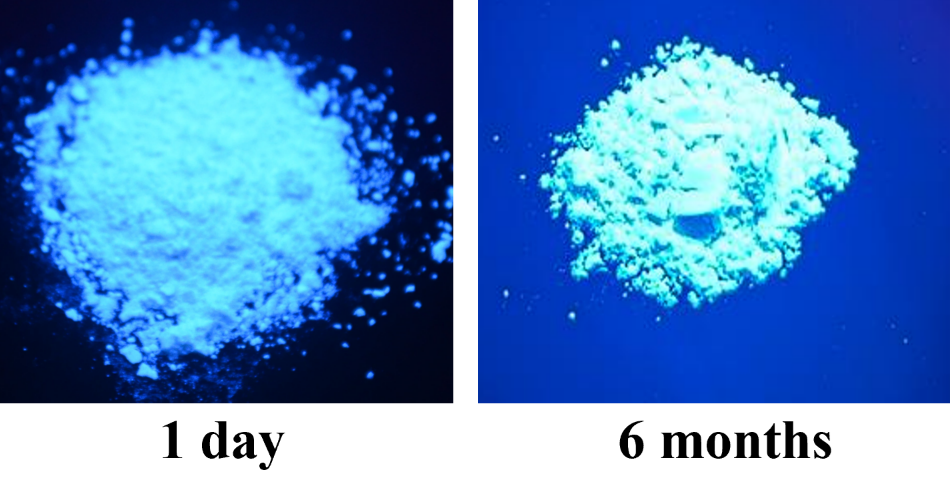

Supplement: S9 Fig — (TIF) [file pone.0296270.s009.tif]

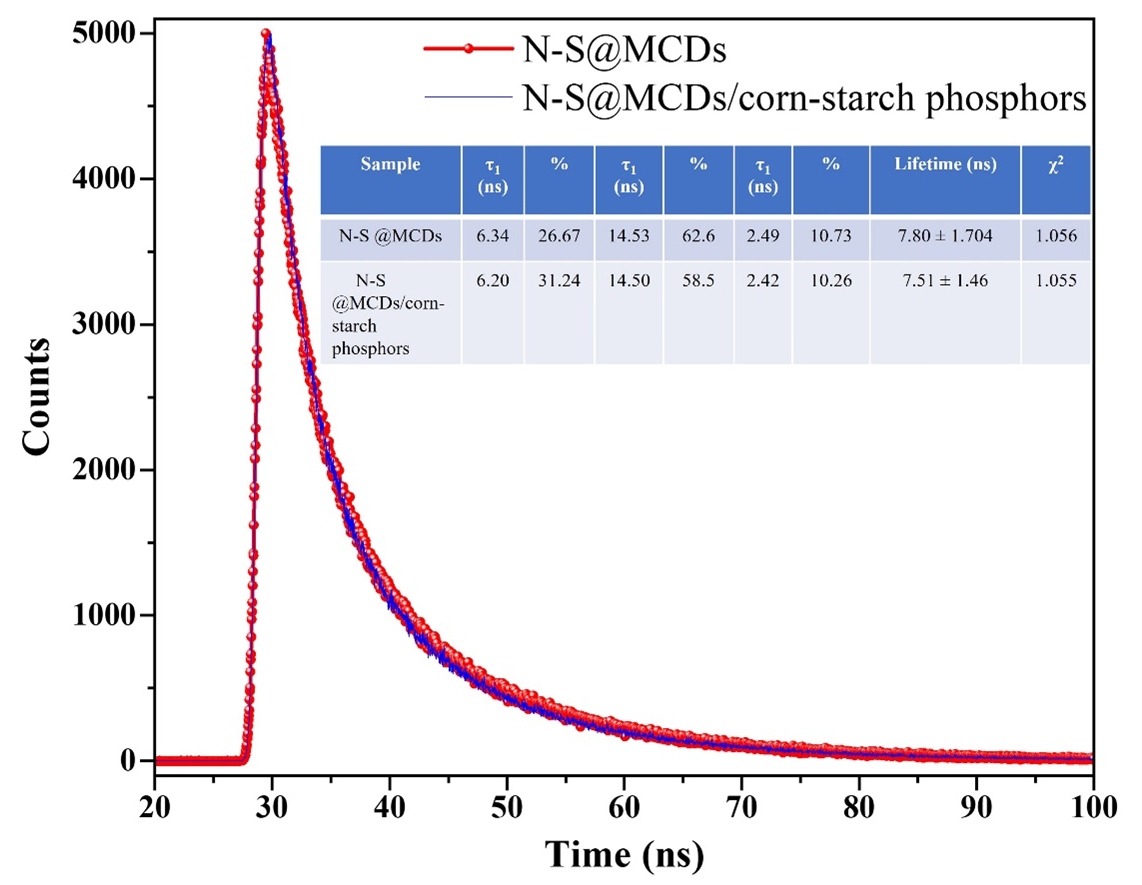

Supplement: S10 Fig — (TIF) [file pone.0296270.s010.tif]
