# Supplementary material for: Harnessing fluorescent carbon quantum dots from natural resource for advancing sweat latent fingerprint recognition with machine learning algorithms for enhanced human identification
Source: PLoS One. 2024 Jan 4;19(1):e0296270. doi: 10.1371/journal.pone.0296270 (PMC10766178; doi:10.1371/journal.pone.0296270)
Supplement: S1 Table — (DOCX) [file pone.0296270.s011.docx]

**S1 Table**. Elemental analysis of N-S@MCDs and N-S@MCDs/corn-starch phosphors.

| **Element** | **Atomic percentage (%)** | |
| --- | --- | --- |
|  | **N-S@MCDs** | **N-S@MCDs/corn-starch phosphors** |
| Carbon | 40.72 | 48.3 |
| Nitrogen | 40.49 | 36.25 |
| Sulfur | 16.6 | 15.05 |
| Oxygen | 2.19 | 0.39 |
| C/N ratio | 1.01 | 1.33 |
| C/S ratio | 2.45 | 3.20 |
