# Supplementary material for: Harnessing fluorescent carbon quantum dots from natural resource for advancing sweat latent fingerprint recognition with machine learning algorithms for enhanced human identification
Source: PLoS One. 2024 Jan 4;19(1):e0296270. doi: 10.1371/journal.pone.0296270 (PMC10766178; doi:10.1371/journal.pone.0296270)
Supplement: S2 Table — (DOCX) [file pone.0296270.s012.docx]

**S2Table.** Green precursor-derived CQDs and their related quantum yield (QY)

| **S. No.** | **Greener source** | **Quantum Yield (%)** | **Ref.** |
| --- | --- | --- | --- |
| 1 | Mango | 0.48-3.92 | [1] |
| 2 | Soy milk | 2.6 | [2] |
| 3 | Rose petals | 9.6 | [3] |
| 4 | Coffee grounds | 3.8 | [4] |
| 5 | Chicken egg | 6-8 | [5] |
| 6 | Waste Frying oil | 3.66 | [6] |
| 7 | Temple waste (marigold) | 11.78 | **Present work** |
