# Supplementary material for: Harnessing fluorescent carbon quantum dots from natural resource for advancing sweat latent fingerprint recognition with machine learning algorithms for enhanced human identification
Source: PLoS One. 2024 Jan 4;19(1):e0296270. doi: 10.1371/journal.pone.0296270 (PMC10766178; doi:10.1371/journal.pone.0296270)
Supplement: S1 Graphical abstract — (PDF) [file pone.0296270.s013.pdf]

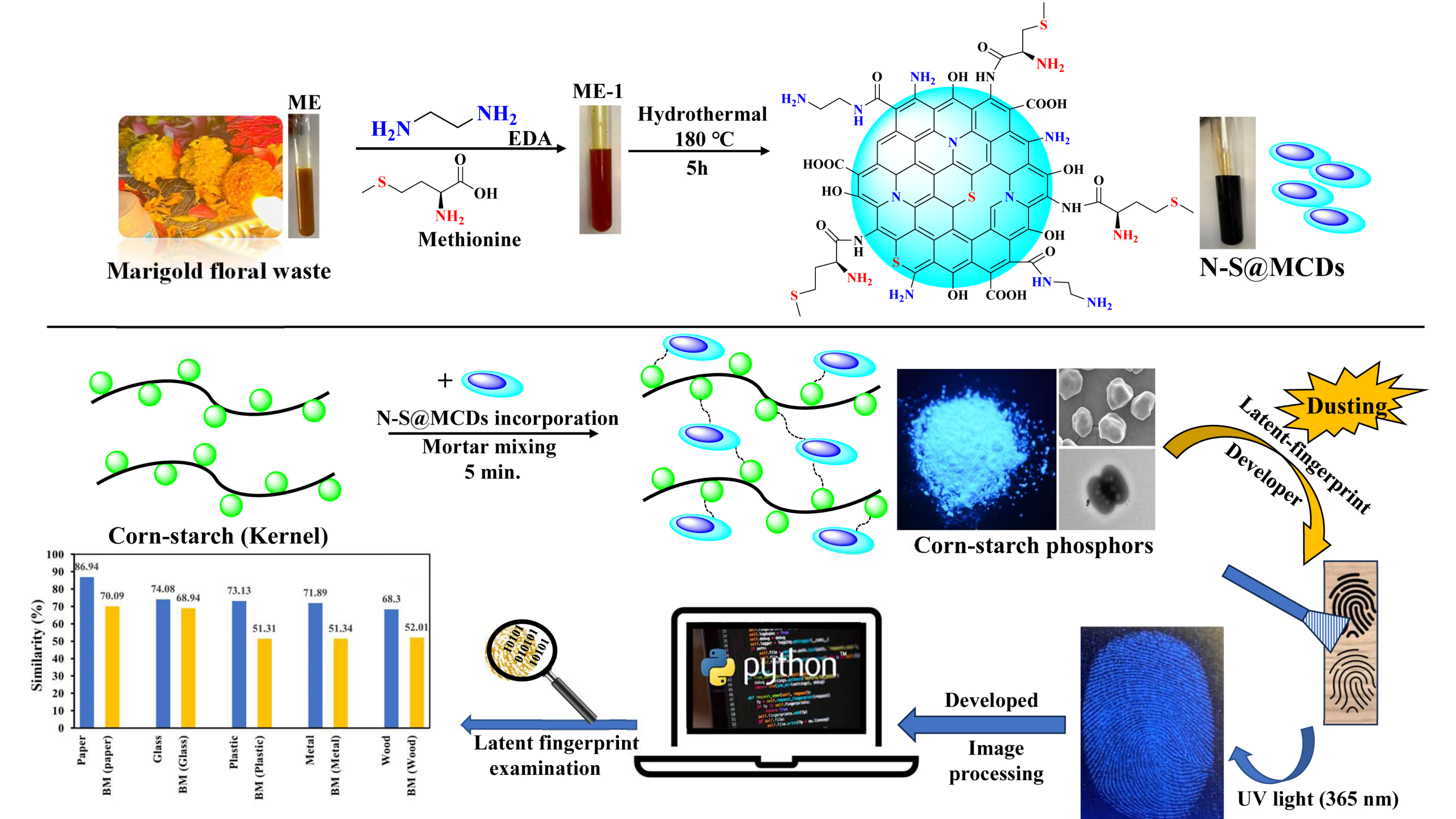

ME
Marigold floral waste
H_2N NH_2
EDA
Methionine
ME-1
Hydrothermal
180 °C
5h
N-S@MCDs
Corn-starch (Kernel)
+
N-S@MCDs incorporation
Mortar mixing
5 min.
Corn-starch phosphors
Dusting
Latent-fingerprint
Developer
UV light (365 nm)
Developed
Image
processing
python
Latent fingerprint
examination
Similarity (%)
86.94
70.09
74.08
68.94
73.13
51.31
71.89
51.34
68.3
52.01
Paper
BM (paper)
Glass
BM (Glass)
Plastic
BM (Plastic)
Metal
BM (Metal)
Wood
BM (Wood)
