# Supplementary material for: Harnessing fluorescent carbon quantum dots from natural resource for advancing sweat latent fingerprint recognition with machine learning algorithms for enhanced human identification
Source: PLoS One. 2024 Jan 4;19(1):e0296270. doi: 10.1371/journal.pone.0296270 (PMC10766178; doi:10.1371/journal.pone.0296270)
Supplement: Attachment — Submitted filename: reviewers response.docx [file pone.0296270.s014.docx]

The authors utilized biomass to prepare the carbon dots for fingerprint applications. The experimental work was well-planned and written systematically. The manuscript can be accepted after doing the following corrections.

1. Please refine the language carefully
2. Please check the section numbers.
3. Align the table 1 properly.
4. Improve the quality of all figures.
