# Supplementary material for: Harnessing fluorescent carbon quantum dots from natural resource for advancing sweat latent fingerprint recognition with machine learning algorithms for enhanced human identification
Source: PLoS One. 2024 Jan 4;19(1):e0296270. doi: 10.1371/journal.pone.0296270 (PMC10766178; doi:10.1371/journal.pone.0296270)
Supplement: Attachment — Submitted filename: comments.docx [file pone.0296270.s015.docx]

Introduction

1. Driving inspiration from nature to take design cues for the preparation of functional materials, to address the grave issues facing the sustainable production of nanomaterials without employing harmful substances, is imperative to use an environmentally benign and renewable raw material source. Carbon quantum dots (CQDs) are a greener example of a functionalized material produced from processing biomass with a distinctive natural composition with fascinating features. The field of naturally occurring CQDs has expanded tremendously; nevertheless, due to inadequate characterization of green precursors, flexible and heterogeneous in nature, the synthesis methodology, and repeatability remain a bottleneck. Utilizing edible green resources for the synthesis of CQDs, such as tomato, banana, milk, orange, cabbage, corn bran, and coconut, has recently attracted a lot of attention[

The sentence formation is not proper and neither linked properly. It is written poorly. Choices of words are inadequate.

What is green resources? It should be replaced with adequate word. This word has been repeated so many times.

2. The majority of CQDs formed from biomass are blue-emitting.

Incomplete sentence.

3. To obtain good mobility and avoid dye powder stagnation and substrate adhesion, these dyes are indeed dispersed in a significant volume of media (powder carrier) in practical applications. Based on the coffee ring effect and the unquenched CQDs fluorescence during the drying process, we also revealed a spraying technique that induces LFPs on impregnable surfaces by utilizing CQDs.
4. In this work, we developed a nano-carbogenic fluorescent fingerprint powder composition (NS@MCDs/corn-starch phosphors) for developing LFPs. Such powder comprises environment friendly highly fluorescent carbon quantum dots (N-S@MCDs) synthesized from biomass (marigold extract). These N-S@MCDs, when incorporated on the corn starch powder using a greener approach, enables the development of highly fluorescent fingerprint patterns on various non-porous surfaces, their quick intensification under UV light to display a brilliant, sharp fingerprint, and their long-term preservation. We used a digital-processing tool to accurately detect LFPs, which retrieved the fine characteristics of LFPs on various substrates, a precise comparison of the characteristics with the control, and then displayed the corresponding scores on a computer. The highest possible matched score was 86.94%. Moreover, we elucidate the conversion mechanism of marigold extract to N-S@MCDs by analyzing the composition of intermediates formed. Translating these promising research findings into real-world technologies with substantial social and economic relevance, necessitates a collaborative, openminded approach involving materials scientists, biologists, and forensic investigators.

**2. Experimental Section**

**Synthesis of hetero-atom doped carbon quantum dots (N-S@MCDs)**

5. After being lyophilized obtained sticky black product, the resulting supernatant (N-S@MCDs) was appropriately redispersed in water.

Sentence formation

**2.2.2. Preparation of N-S@MCDs/corn-starch phosphors**

6. N-S@MCDs and corn-starch are mixed in a mortar followed by the addition of Milli Q water as a binder by ultrasonic agitation over 10 min at RT

Check Grammar

**2.3. Characterization and Instrumentations**

7. **Morphology.** The high-resolution X-ray diffraction (XRD) pattern was produced using a Cu K radiation source and a PANalytical Empyrean X-ray diffractometer at an acceleratingpotential of 40 kV, in the 2θ range of 10-60° with a Cu Kα radiation source. X-rays were acquired of solid samples that had been finely powdered. Transmission electron microscopy (TEM) and high-resolution transmission electron microscopy were used to assess the surface morphology and particle size of N-S@MCDs (HRTEM). TEM images were obtained on a

JEOL JEM-2100F electron microscope with a 200 kV acceleration voltage. The material was

drop-cast onto a copper grid that had been coated with carbon, and then the samples were

allowed to dry at room temperature. Scanning Electron Microscope (SEM) with EDAX

(Energy-dispersive X-ray) spectroscopy was done from the University of Delhi using Jeol JSM 6610LV using Tungsten electron sources at 10KV with Magnification X5 to X 3,00,000 with High Vacuum mode using LN2 free EDS detector. 1H and 13C NMR (400 MHz for proton and 100 MHz for carbon) spectra were recorded with a JEOL spectrometer using trimethylsilane as an internal standard.

8. The associated d-spacing of N-S@MCDs is higher than the bulk graphite i.e., 3.34 Å, which depicts turbostratic carbon structure (sp2 deformation). Additionally, one more peak centered around 42.50° (2θ) represents a predominantly graphitic structure with an interlayer spacing of 0.21 nm[26]. Moreover, TEM and HR-TEM images were utilized to examine particle shape, size, and nature of N-S@RCD. Fig. 3[a-f] shows that as-prepared N-S@MCDs were uniform, mono-dispersed spheres with a mean diameter of 6.4±1.64 nm

Page 14- variation in font

9. The particle size distribution of assynthesized N-S@MCDs is shown in **Fig. S1**, based on 50 counts. The HR-TEM image validates the N-spherical S@RCD's form, and it also shows some particles to have well resolved lattice fringes (0.35 nm, d-spacing).

**Where is fig. S1??**

10. Additionally, the EDAX spectrum confirms the existence of C, N, O and S in N-S@MCDs and NS@ MCDs/corn-starch phosphors (Fig. S2 and Fig. S3, respectively). Elemental analysis of NS@ MCDs and N-S@MCDs-coated corn-starch was carried out and the results were consolidated in Table S1.

(Page 17)

11. Typically, there are three levels in which fingerprint patterns can be classified. Level 1 displays whorl, loop and delta which were not distinct enough to recognized. However, the level two feature points, which are well-known for providing the most crucial identifying data in fingerprint analysis by assigning uniqueness and invariance to fingerprints through random combinations. Level 2 features refer to minute points like islands, lakes, hooks, short ridges, spur, eye, core, crossover, trifurcations, and bifurcations that were obviously recognizable patterns.

**Explain level 1 and whether level 2 is minutiae points or minute point??**

12. The fact that the improved LFPs clearly show different ridge details without background interference shows how adaptable, ubiquitous, and realistically helpful our visualization technique is for improving LFPs on nearly all porous and non-porous surfaces.

Page 25- rewrite

13. Python digital processing has grown to be a crucial tool for evaluating LFPs pictures as one of the artificial intelligence approaches [54]. This approach is based on the minute characteristics that are created by a machine learning programme from the local information points inherent in fingerprint scans. The original LFPs image should be distinct enough, which indicates that it has distinct level 1 and level 2 feature structures. The fingerprint's original picture is transformed into a binary model in three phases prior to extracting feature points [8,9,54,55]. Firstly, a grayscale picture is created from the input color image (Fig. 12). To emphasize the target contour, the picture is then normalized. Finally, binaryzation is accomplished by classifying various pixels into "1" or "0" categories based on their grayscale values, that is, the image's pixels are redrew as either white (1) or black (0). After binaryzation, the noisy (blur) areas get sharper while the noise spots and shadows vanish (Fig. 12). Additionally, Fig. 12 provides further information about the level 2 and 3 LFPs.

Not explained properly.

grammar

level 3 characteristics are?
